# Ursolic Acid and Its Nanoparticles Are Potentiators of Oncolytic Measles Virotherapy against Breast Cancer Cells

**DOI:** 10.3390/cancers13010136

**Published:** 2021-01-04

**Authors:** Ching-Hsuan Liu, Shu Hui Wong, Chen-Jei Tai, Cheng-Jeng Tai, Yu-Chi Pan, Hsue-Yin Hsu, Christopher D. Richardson, Liang-Tzung Lin

**Affiliations:** 1 Graduate Institute of Medical Sciences, College of Medicine, Taipei Medical University, Taipei 110, Taiwan; d119107007@tmu.edu.tw; 2 Department of Microbiology & Immunology, Dalhousie University, Halifax, NS B3H 4R2, Canada; chris.richardson@dal.ca; 3 International M.Sc. Program in Medicine, College of Medicine, Taipei Medical University, Taipei 110, Taiwan; m142107001@tmu.edu.tw; 4 Department of Traditional Chinese Medicine, Taipei Medical University Hospital, Taipei 110, Taiwan; chenjtai@tmu.edu.tw; 5 Department of Obstetrics and Gynecology, School of Medicine, College of Medicine, Taipei Medical University, Taipei 110, Taiwan; 6 Division of Hematology and Oncology, Department of Internal Medicine, Taipei Medical University Hospital, Taipei 110, Taiwan; cjtai@tmu.edu.tw; 7 Department of Internal Medicine, School of Medicine, College of Medicine, Taipei Medical University, Taipei 110, Taiwan; 8 Department of Microbiology and Immunology, School of Medicine, College of Medicine, Taipei Medical University, Taipei 110, Taiwan; rita10732@gmail.com; 9 Department of Life Sciences, Tzu-Chi University, Hualien 970, Taiwan; hsueyin@mail.tcu.edu.tw; 10 Department of Pediatrics and Canadian Center for Vaccinology, Izaak Walton Killam Health Centre, Halifax, NS B3K 6R8, Canada

**Keywords:** oncolytic virotherapy, measles virus, ursolic acid, nanoparticles, combination treatment

## Abstract

**Simple Summary:**

Despite the advancing treatments, female breast cancer is one of the most common cancers and a leading cause of cancer deaths in women. To help broaden the therapeutic spectrum of breast cancer, we identified the natural compound ursolic acid (UA) as a potentiator that enhances the oncolytic activity of measles virus (MV) against breast cancer cells through the induction of apoptosis. In addition, to increase clinical applicability, we further generated UA nanoparticles that achieved improved solubility. UA nanoparticles similarly synergized with MV in killing breast cancer cells by triggering apoptosis, and this synergistic anticancer effect was also observed in various breast cancer cell types. This study demonstrates for the first time that UA and its nanoparticles enhance MV’s oncolytic activity in breast cancer cells, suggesting that such combinations may be worth further exploring as an anticancer strategy against breast cancer.

**Abstract:**

Oncolytic viruses (OVs) and phytochemical ursolic acid (UA) are two efficacious therapeutic candidates in development against breast cancer, the deadliest women’s cancer worldwide. However, as single agents, OVs and UA have limited clinical efficacies. As a common strategy of enhancing monotherapeutic anticancer efficacy, we explored the combinatorial chemovirotherapeutic approach of combining oncolytic measles virus (MV), which targets the breast tumor marker Nectin-4, and the anticancer UA against breast adenocarcinoma. Our findings revealed that in vitro co-treatment with UA synergistically potentiated the killing of human breast cancer cells by oncolytic MV, without UA interfering the various steps of the viral infection. Mechanistic studies revealed that the synergistic outcome from the combined treatment was mediated through UA’s potentiation of apoptotic killing by MV. To circumvent UA’s poor solubility and bioavailability and strengthen its clinical applicability, we further developed UA nanoparticles (UA-NP) by nanoemulsification. Compared to the non-formulated UA, UA-NP exhibited improved drug dissolution property and similarly synergized with oncolytic MV in inducing apoptotic breast cancer cell death. This oncolytic potentiation was partly attributed to the enhanced autophagic flux induced by the UA-NP and MV combined treatment. Finally, the synergistic effect from the UA-NP and MV combination was also observed in BT-474 and MDA-MB-468 breast cancer cells. Our study thus highlights the potential value of oncolytic MV and UA-based chemovirotherapy for further development as a treatment strategy against breast cancer, and the feasibility of employing nanoformulation to enhance UA’s applicability.

## 1. Introduction

Female breast cancer is the most commonly diagnosed adenocarcinoma globally [1], accounting for 1.7 million new cases per year and 25% of all cancers [2]. Despite advances in the currently available therapies, metastatic breast cancer remains incurable with high mortality rate [3], making breast cancer the second leading cause of all cancer deaths and top among cancer deaths in women [4]. Efficacies of conventional therapies are limited by issues of poor tumor specificity, off-target toxicities, low drug bioavailability, rapid drug clearance, and incomplete tumor eradication, which can potentially be overcome by utilizing nanomedicine-based therapeutic approaches [5,6]. Nanomedicine is instated as a key driver of modern clinical advancements, largely defined as the application of nanoscale agents (<1000 nm) for diagnosis or treatment of diseases [5,6]. Their small size and capacity for surface and/or intraparticulate modifications allow them to exhibit superior targeting, safety, solubility, bioavailability, and potency [5], which are crucial for efficacious anticancer therapies. Thus, cancer nanomedicines, including nanoparticle drug formulation and oncolytic viruses (OVs), have been extensively studied, with clinical success attained for multiple cancers including breast cancer [5].

Nanoparticle drug formulation is a popular strategy as it efficiently improves in vivo drug solubility, bioavailability, and bioactivity, especially for phytocompounds [7], a safe and cost effective source of drug development. Widely found in fruits and plants, ursolic acid (UA; 3-β-hydroxy-urs-12-en-28-oic acid) is a naturally occurring pentacyclic triterpenoid recognized for its significant multimodal anticancer properties against various cancer types [8,9]. As a key candidate in recent studies investigating chemopreventive and chemotherapeutic agents for breast cancer, it was found to inhibit breast cancer proliferation, angiogenesis, metastasis, and induce apoptosis both in vitro and in vivo [10]. Nonetheless, like many phytocompounds, UA suffers poor solubility and bioavailability in vivo [10], which can be effectively circumvented by nanotechnology modifications such as nanoparticle formulation.

The use of OVs as cancer nanomedicine, oncolytic virotherapy, is another promising therapeutic strategy that selectively targets and destroys cancer cells [11], via direct killing of infected cancer cells and induction of tumor-specific immunity against cancer recurrence [12]. Among the oncolytic viral agents explored against breast cancer [13], measles virus (MV) recently emerged as an important OV. The enveloped, negative-sense, single-strand RNA virus utilizes three host cell receptors for infection, including the membrane cofactor protein CD46 (cluster of differentiation 46), signaling lymphocytic activation molecule (SLAM; or CD150), and the tumor marker Nectin-4 (or poliovirus receptor related protein 4; PVRL4) overexpressed in adenocarcinomas [14,15,16,17,18,19]. Laboratory/vaccine strains of MV can utilize all three receptors, while wild type strains only employ SLAM and Nectin-4. Due to the usage of Nectin-4 tumor marker and not the ubiquitously expressed CD46 receptor for infection, wild type strain MV has gained attention as a potentially suitable targeted oncolytic agent. It is possible to engineer Nectin-4-specific MV to exclusively target and destroy Nectin-4-positive breast tumors and other adenocarcinomas [20]. Alternatively, future development can include attenuating the wild type strain-based oncolytic MV vector by removing the C and V proteins expression [21] to enhance its safety for administration to human patients. Therefore, in the current study, we use the wild type strain MV that is more specific to Nectin-4 as a proof of concept for wild type MV’s suitability as a targeted oncolytic vector against Nectin-4-positive tumors.

Despite excellent safety profile and preclinical anticancer potential, many oncolytic viruses including MV exhibit modest efficacy as a single agent [22]. Combinatorial chemovirotherapy in which oncolytic viruses are combined with an anticancer compound is thus commonly used to enhance anticancer efficacy of virotherapeutics. Oncolytic MV and the phytochemical UA could conceivably enhance cytotoxicity against breast cancer cells due to their distinct mechanisms of anticancer activities. This potentially enables better tumor killing while preventing development of resistance [23]. As a proof-of-concept, the present study focuses on exploring the anticancer potency of a chemovirotherapy consisting of recombinant wild type MV plus UA or its nanoparticles as a novel strategy for breast cancer.

## 2. Results

### 2.1. UA and Oncolytic MV Exhibit Anticancer Activity against MCF-7 Breast Cancer Cells

**Figure 1:**
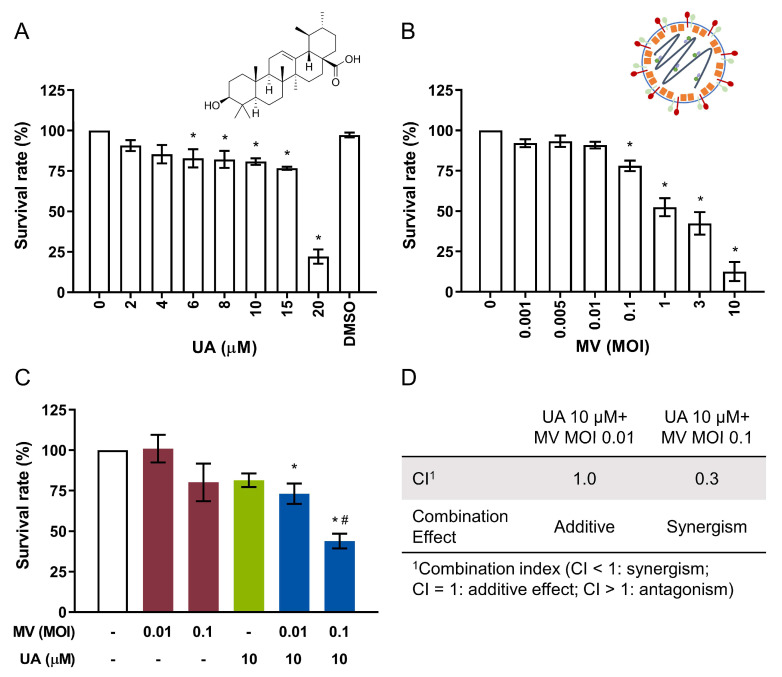
Ursolic acid (UA) and oncolytic measles virus (MV) are cytotoxic to human breast cancer MCF-7 cells and synergistically induce anticancer activity. (**A**) MTT cell viability analysis of MCF-7 cells treated with UA (2–20 µM) for 5 days. (**B**) MTT cell viability analysis of MCF-7 cells treated with MV (multiplicity of infection, MOI 0.001–10) for 5 days. (**C**) MTT cell viability analysis of MCF-7 cells treated with UA (10 µM) and MV (MOI 0.01 or 0.1) concurrently for 5 days. (**D**) Analysis of treatment synergism using the Chou–Talalay method wherein combination index (CI) value quantitatively defines synergism (CI < 1), additive effect (CI = 1), or antagonism (CI > 1). All data shown are means ± SEM from three independent experiments; * *p* < 0.05 in (**A**,**B**) compared to ‘0’; * *p* < 0.05 compared with MV MOI 0.01 or 0.1, ^#^ *p* < 0.05 compared with UA treatment only in (**C**); DMSO = 0.2% (the maximum concentration of DMSO used).

We first examined the individual anticancer effect of UA and oncolytic MV on human breast cancer cell line MCF-7 by testing a range of drug concentrations or viral multiplicity of infection (MOI) for 5 days and evaluating their effects on cell viability. UA was prepared in DMSO solvent as it is insoluble in water. Following UA treatment, cell viability decreased gradually to 75% as UA concentration increased from 2 to 15 µM, and 20 µM UA sharply reduced cell viability to 20% (Figure 1A). The 50% cytotoxic concentration (CC_50_) value of UA was 16.67 ± 1.10 µM for the 5-day treatment. As for oncolytic MV, which is known to highly infect MCF-7 cells due to their upregulated Nectin-4/PVRL4 expression [24], cell viability exhibited dose-dependent decrease over the range of MOIs tested (0.001 to 10) (Figure 1B). Infection with the oncolytic MV at MOI of 1 decreased cell viability by about 50%. Based on these results, UA and MV were thus used at concentrations below their CC_50_ indices (≤10 µM for UA and MOI 0.1–0.01 for MV) for the subsequent experiments devised for exploring their potential synergistic activities.

### 2.2. Combined Treatment of UA and Oncolytic MV Produces Synergistic Anticancer Effect against MCF-7 Breast Cancer Cells

To determine whether UA and oncolytic MV would exert stronger potency when used in combination, both agents were concurrently added to MCF-7 cells. Data obtained from the cell viability analysis in the ensuing incubation was then assessed by the Chou–Talalay method [25], where the combination index (CI) value would signify the combination effect to be synergistic (CI < 1), additive (CI = 1), or antagonistic (CI > 1). While the combination of 10 µM UA with MOI 0.01 of oncolytic MV attained a similar killing effect as UA mono-treatment (~25%) with no obvious synergism, increasing the viral concentration to MOI 0.1 with 10 µM UA produced a significantly higher MCF-7 cell death (>50%) compared to each agent alone (Figure 1C). The CI value of the MV MOI 0.1 with 10 µM UA combination was 0.3 (Figure 1D), indicating that UA and oncolytic MV can act synergistically, leading to enhanced anticancer effect against MCF-7.

### 2.3. UA Treatment Does Not Antagonize Oncolytic MV Infection

**Figure 2:**
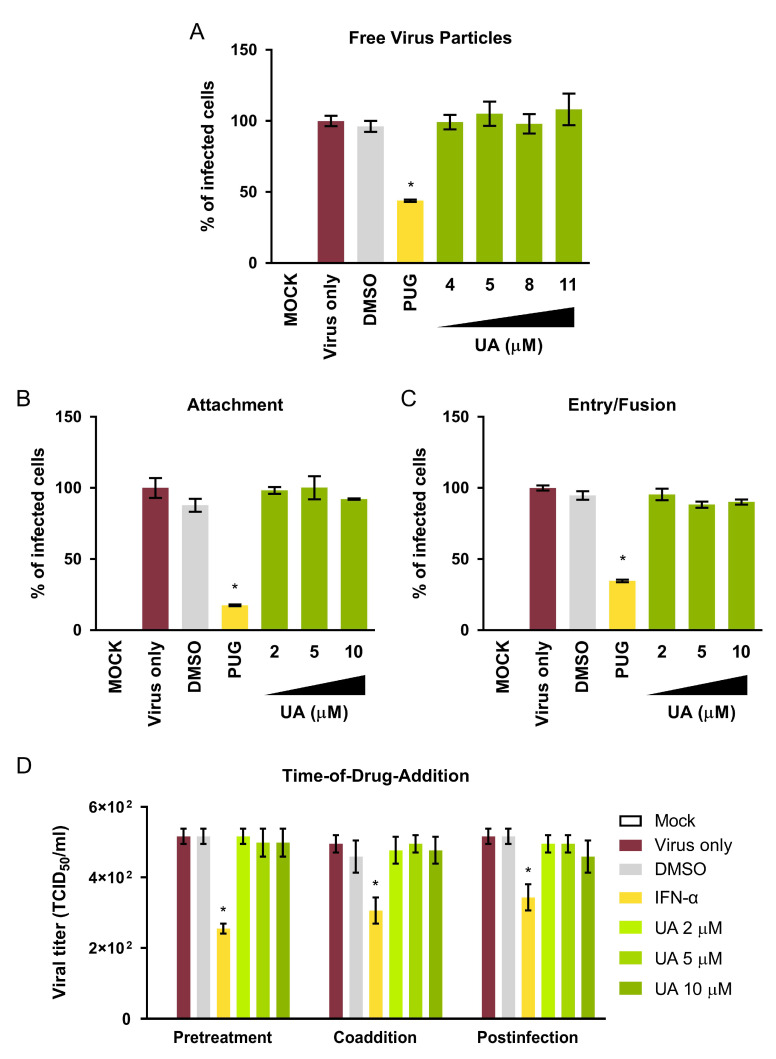
UA treatment does not interfere with the infection of oncolytic MV. (**A**) Effect of UA (4, 5, 8 and 11 µM) on free MV particles. (**B**) Effect of UA (2, 5 and 10 µM) on MV attachment. (**C**) Effect of UA (2, 5 and 10 µM) on MV entry/fusion. (**D**) Time-of-drug-addition analysis of UA treatment on MV infection. For (**A**–**C**), viral reporter fluorescence intensity reflecting the infectivity was measured using a variable mode scanner at 72 h post-infection. For (**D**), supernatants from the experiment were collected at 72 h post-infection for viral titration using 50% tissue culture infective dose (TCID_50_). For all assays, final MV concentration = MOI 0.1; DMSO = 0.1% (the maximum concentration of DMSO used); 50 µM punicalagin (PUG) for (**A**–**C**) or 1000 IU/mL interferon-α (IFN-α) for (**D**) was included as a positive control. All data shown are means ± SEM from three independent experiments; * *p* < 0.05.

While UA and MV co-treatment demonstrated synergy, precaution was taken to further examine whether UA would interfere with the oncolytic MV infection by evaluating its effect on the viral infectivity through a series of experiments, each focused on a specific stage of MV infection in MCF-7. For the early viral entry stages, three assays were performed to assess the impact of UA on (i) free oncolytic MV particles (Figure 2A), (ii) viral attachment to the target cells (Figure 2B), and (iii) post-attachment fusion with the target cells (Figure 2C). A known small-molecule MV entry inhibitor punicalagin (PUG) [26] was included as a positive control in all experiments. Data obtained from viral reporter fluorescence showed that UA, at concentrations up to the maximum dose used in the combination treatment, did not affect the early entry steps of the oncolytic MV, similar to the DMSO solvent control. PUG, on the other hand, effectively impeded all three steps as previously reported [26]. Three time-of-drug-addition assays (pre-treatment, co-addition, and post-infection) were also performed to assess whether UA treatment administered at different time-points would produce antiviral effect on the oncolytic MV infection. UA treatment generally had negligible effects on the MV infectivity for all doses tested, whereas the positive control interferon-α (IFN-α) significantly reduced the viral infection (Figure 2D). These results therefore suggested that combinatorial treatment of UA and the oncolytic MV does not negatively modulate the viral infection.

### 2.4. UA and Oncolytic MV Combinatorial Treatment Enhances Apoptotic Cell Death of MCF-7 Breast Cancer Cells

**Figure 3:**
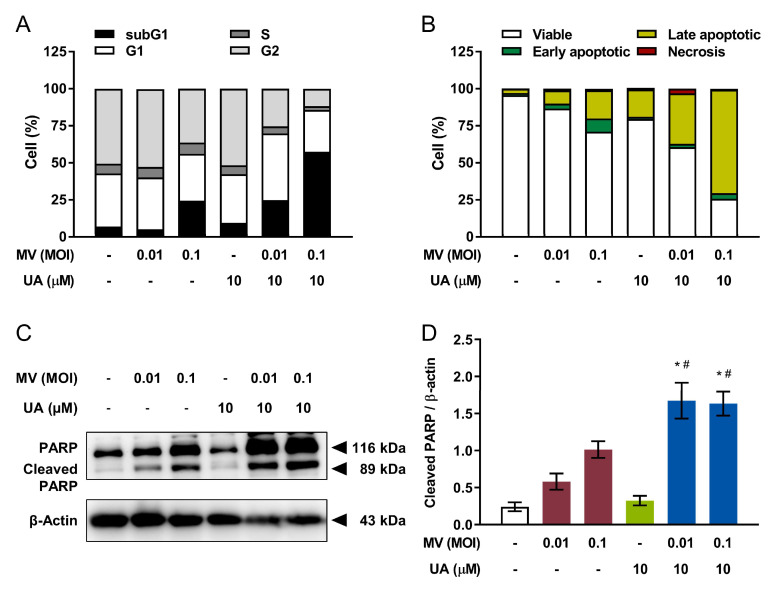
Co-treatment using UA and oncolytic MV enhances apoptotic cell death in human breast cancer MCF-7 cells. MCF-7 cells were first treated with combination of UA (10 µM) and MV (MOI 0.01 or 0.1) for 5 days, then analyzed by flow cytometry for (**A**) cell cycle distribution and (**B**) apoptosis induction, using propidium iodide (PI) staining and double staining (PI and Annexin V conjugated with allophycocyanin [APC]) respectively. Percentages shown are determined by Beckman Cytomics TM FC500 Flow Cytometry CXP analysis software. (**C**) Lysates of MCF-7 cells co-treated with UA (10 µM) and MV (MOI 0.01 or 0.1) for 5 days were analyzed by western blot for poly (ADP-ribose) polymerase (PARP) cleavage. (**D**) Quantitative analysis of the relative level of cleaved PARP from (**C**). All quantitative data are expressed as means ± SEM from three independent experiments; * *p* < 0.05 compared with MV MOI 0.01 or 0.1, ^#^ *p* < 0.05 compared with UA treatment only.

Cell cycle and apoptosis analyses were next performed to study the mechanism underlying the anticancer activity of UA and oncolytic MV combinatorial treatment on MCF-7 cells. Compared to cells treated with UA or MV alone, the combinatorial treatments caused synergistic elevation in the population of sub-G1 phase cells to approximately 25% and 60% for MOI 0.01 and MOI 0.1 of oncolytic MV, respectively (Figure 3A). Increased sub-G1 population suggesting apoptosis [27] was subsequently validated by flow cytometric Annexin V/Propidium iodide (PI) double staining. Percentage of late apoptotic cells detected in MCF-7 cells treated with UA and MV in combination significantly increased to 33–69% compared to 20% or less when treated with each agent alone (Figure 3B). These results were further confirmed via western blot analysis of the apoptotic marker, cleavage of poly (ADP-ribose) polymerase (PARP) [28]. Consistently, our finding revealed increased apoptosis as level of cleaved PARP was greatly enhanced by UA and oncolytic MV combinatorial treatments (Figure 3C,D). Overall, our results demonstrated that UA and oncolytic MV combinatorial treatment produces synergistic anticancer effect against MCF-7 breast cancer cells, which is mediated by increased induction of apoptotic cell death.

### 2.5. Nanoformulation Changes the Physicochemical Properties and Improves Drug Dissolution of UA

**Figure 4:**
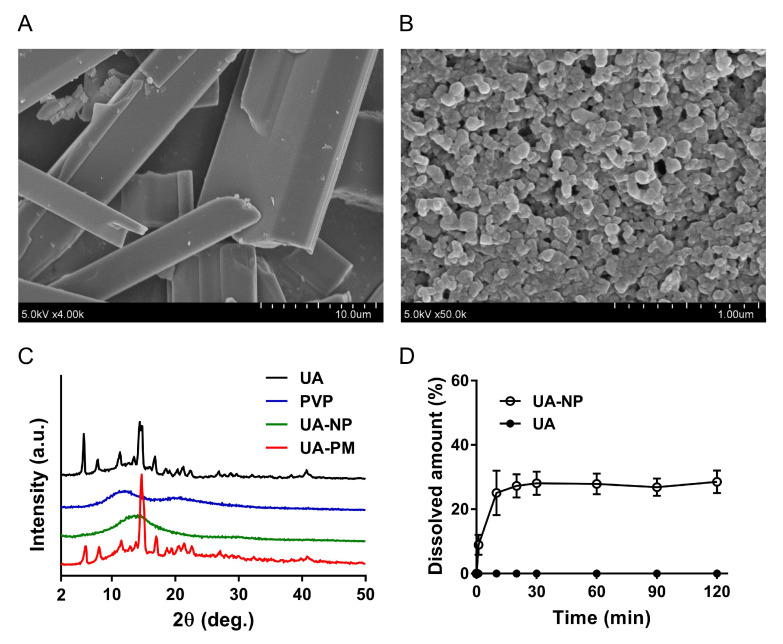
Physicochemical properties of UA nanoparticles (UA-NP). (**A**) Field emission scanning electron microscopy (FESEM) photograph of non-formulated UA (magnification: 4000×; scale bar = 10.0 μm). (**B**) FESEM photograph of nanoformulated UA (magnification: 50,000×; scale bar = 1.0 μm). (**C**) X-ray diffraction patterns of UA, polyvinylpyrrolidone (PVP), nanoformulated UA nanoparticles (UA-NP), and UA-PVP physical mixture (UA-PM). XRD patterns were taken from 2° to 50° with a scanning rate of 4°/min. The spectra were offset for clarity. (**D**) Drug dissolution test (open circle, UA-NP; filled circle, UA). Samples containing 4.68 mg equivalent of UA were placed in 100 mL of pH 7.4 phosphate buffer and maintained at 37 ± 0.5 °C. During the dissolution process, samples were withdrawn at 0, 1, 10, 20, 30, 60, 90 and 120 min for HPLC analysis. Data points are expressed as means ± SD (*n* = 6).

As UA is a triterpenoid compound with poor water solubility and low bioavailability in vivo, which limit its applicability, we next employed nanoemulsification using the nonionic polymer polyvinylpyrrolidone (PVP) to generate water soluble PVP-based UA nanoparticles (UA-NP) as a strategy to improve these issues [7]. Physicochemical characterization of UA and UA-NP were performed and documented in Figure 4. Our optimized UA:PVP formulation (1:1 weight ratio) produced a well-suspended colloidal solution, while other formulations with 1:3 or 1:6 weight ratio were unable to maintain the dispersion state and easily aggregate or precipitate (data not shown). The mean size of the formulated UA-NP was 209.3 ± 1.7 nm, and the yield was 69.3 ± 9.9% after removing the non-formulated aggregates. Field emission scanning electron microscopy (FESEM) demonstrated a morphological change of the needle-shaped UA crystals (Figure 4A) to the nanoparticulate form of UA-NP (Figure 4B). This observation was supported by the X-ray diffraction (XRD) analysis, in which the obvious crystalline peaks in the spectra of non-formulated UA and the UA-PVP physical mixture (UA-PM) disappeared in the spectra of UA-NP that showed an amorphous state similar to PVP (Figure 4C). This physicochemical change is a favorable factor for improving UA’s dissolution and thus bioavailability [29]. Our dissolution test further confirmed that UA-NP’s solubility was substantially increased over the time intervals tested as compared to non-formulated UA, which remained mostly insoluble in the water-based buffer (Figure 4D). Altogether, these results demonstrated that we have successfully generated nanoformulated UA with improved solubility.

### 2.6. UA-NP Retained Synergistic Anticancer Effect in Combination with Oncolytic MV and Enhanced Apoptotic Cell Death in MCF-7 Breast Cancer Cells

**Figure 5:**
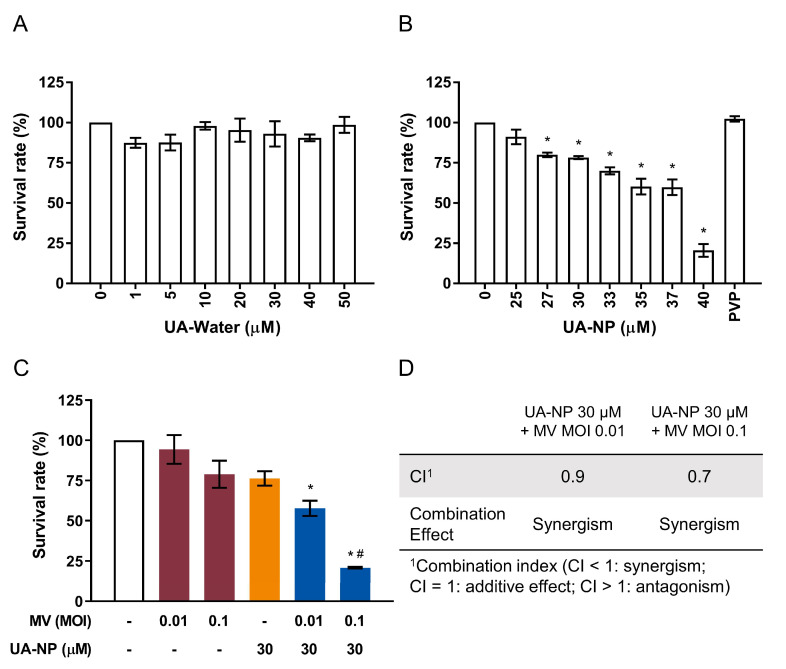
UA-NP retain cytotoxic activity against human breast cancer MCF-7 cells and exert synergistic anticancer activity in combination with oncolytic MV. MCF-7 cells were treated with (**A**) non-formulated UA mixed in water (UA-Water; 1–50 µM) or (**B**) UA-NP solubilized in water (25–40 µM) for 5 days, and cell viability was analyzed by MTT assay; PVP = 50 µg/mL. (**C**) MCF-7 cells were concurrently treated with UA-NP (30 µM) and MV (MOI 0.01 or 0.1) for 5 days, following which cell viability was determined by MTT assay. (**D**) Analysis of treatment synergism using the Chou-Talalay method as in Figure 1. All data shown are means ± SEM from three independent experiments; * *p* < 0.05 in (**A**,**B**); * *p* < 0.05 compared with MV MOI 0.01 or 0.1, ^#^ *p* < 0.05 compared with UA treatment only in (**C**).

To confirm whether UA-NP retained its anticancer potency, the nanoparticles were used to treat MCF-7 cells over a range of drug concentrations. For comparison, non-formulated UA mixed in water (“UA-Water”), in which it is not soluble, was also tested in MCF-7 cells. As shown in Figure 5A, the non-soluble UA-water mixture had no impact on the cell viability of the breast cancer cells. In contrast, the UA-NP solubilized in water could dose-dependently reduce MCF-7 cell viability and the CC_50_ value was found to be 36.52 ± 1.02 µM (Figure 5B). The polymeric carrier PVP alone showed no effect in reducing the MCF-7 breast cancer cell viability. To examine whether UA-NP retained the synergistic anticancer effect in combination with oncolytic MV, 30 µM of the UA-NP and oncolytic MV (MOI 0.01 and 0.1) were used to co-treat MCF-7 cells. As shown in Figure 5C, MCF-7 cell viability was significantly reduced with the MV MOI 0.1 plus UA-NP 30 µM combination compared to each agent alone. Similar results to the above observations were obtained using lactate dehydrogenase (LDH) release assay (Supplementary Figure S1). Their corresponding CI values, 0.9 and 0.7 (Figure 5D), indicated that both groups of combinatorial treatment produced synergistic effect (CI < 1) on MCF-7 cells. 

**Figure 6:**
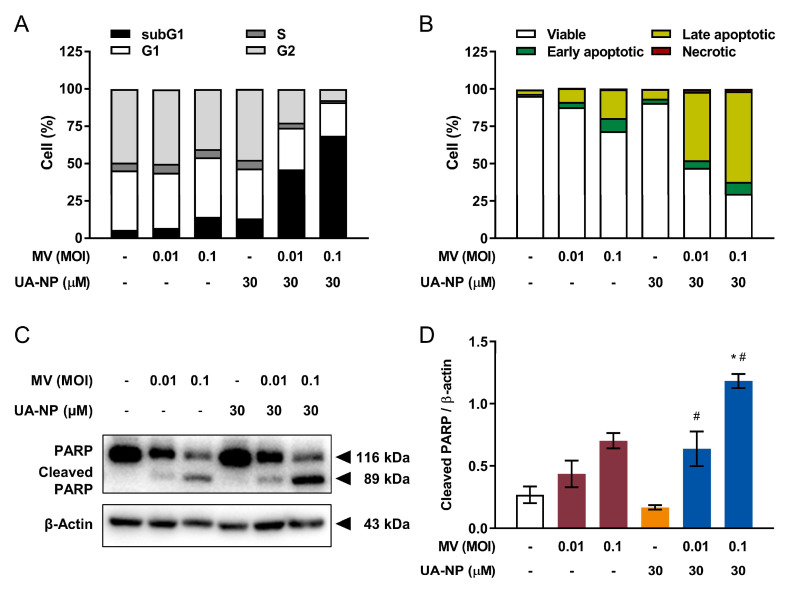
UA-NP and oncolytic MV co-treatment induces enhanced apoptotic cell death in human breast cancer MCF-7 cells. MCF-7 cells were first concurrently treated with UA-NP (30 µM) and MV (MOI 0.01 or 0.1) for 5 days, then analyzed by flow cytometry for (**A**) cell cycle distribution and (**B**) apoptosis induction as in Figure 3A,B. (**C**) Lysates of MCF-7 cells co-treated with UA-NP (30 µM) and MV (MOI 0.01, or 0.1) for 5 days were also analyzed for PARP cleavage by western blot. (**D**) Quantitation of the relative level of cleaved PARP from (**C**). All quantitative data are expressed as means ± SEM from three independent experiments; * *p* < 0.05 compared with MV MOI 0.01 or 0.1, ^#^ *p* < 0.05 compared with UA treatment only.

Finally, cell cycle analysis showed that the combinatorial treatments of UA-NP and oncolytic MV, at MOIs 0.01 and 0.1, similarly enriched sub-G1 phase populations to 46% and 69%, respectively (Figure 6A). With this result predicting increased apoptosis, Annexin V/PI staining likewise demonstrated enhanced levels of apoptosis from below 20% in the untreated and mono-treated cells, to 45–60% in UA-NP and MV co-treated cells (Figure 6B). Markedly higher levels of cleaved PARP from the western blot analysis were also observed in the combinatorial treatments compared to the respective mono-treatment doses, although the MV MOI 0.01 plus UA-NP 30 µM combination did not show a statistical significance compared with MV MOI 0.01 (Figure 6C,D). These observations therefore suggested that UA-NP with improved solubility retain the anticancer activity and can act synergistically with the oncolytic MV through enhancing apoptosis of MCF-7 cells.

### 2.7. Oncolytic MV and UA-NP Combined Treatment Include Autophagic Flux

**Figure 7:**
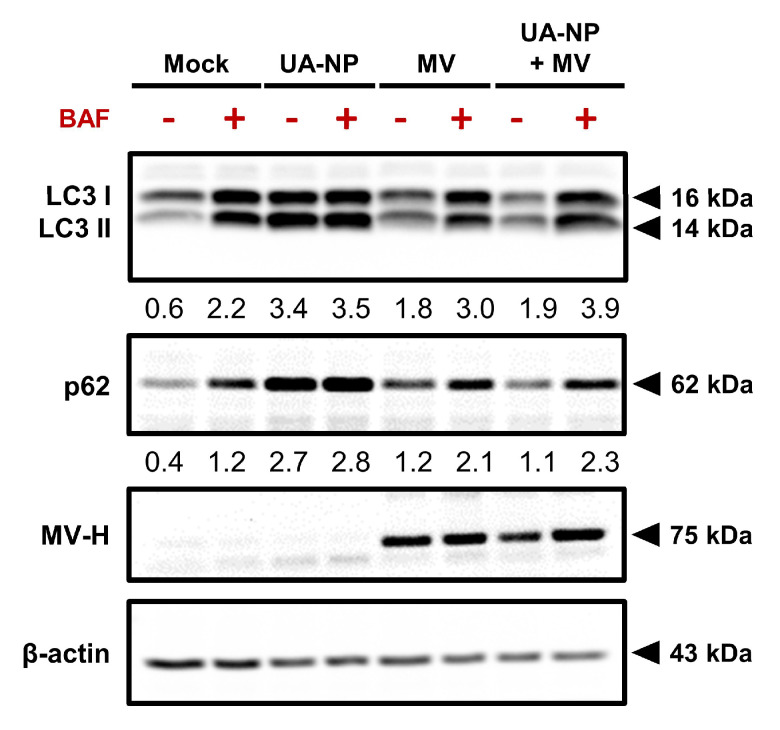
UA-NP and oncolytic MV co-treatment enhances autophagic flux in MCF-7 cells. MCF-7 cells were treated with UA-NP (30 μM), MV (MOI 0.1), or concurrently with both agents for 48 h before being harvested and analyzed for LC3, p62, MV H protein, and β-actin expression using western blot. Bafilomycin (BAF, 100 nM) was added to the indicated groups 4 h before harvesting the cells. LC3II and p62 signals were quantified and normalized to the β-actin loading control.

UA has been reported to induce autophagy in MCF-7 cells at the range of concentrations that we used [30], and interestingly, autophagy also plays a pro-viral role in the life cycle of MV by promoting its replication and particle production [31]. To investigate how the MV and UA-NP combination affects the dynamic process of autophagy in MCF-7 cells, we performed a western blot to analyze the autophagic flux induced by both agents (Figure 7). In contrast to the mock control, which did not induce LC3 lipidation (LC3II, an autophagy marker) at 48 h, UA-NP treatment and MV infection each individually induced autophagy as indicated by the increased LC3II levels. However, the combination of UA-NP and MV led to a decrease in LC3 lipidation compared to UA-NP alone and comparable to MV alone, suggesting enhanced autophagic flux, an event that could be reversed by the lysosomal inhibitor bafilomycin (BAF). This observation in cells that were treated with the combination was also supported by the decreased level of p62, an ubiquitin- and LC3-binding protein that accumulates when autophagy is impaired, such as by BAF treatment [32]. Since enhanced autophagic flux promotes MV’s replicative spread and thus the subsequent cytopathic effect [31], this may contribute to the observed augmented cancer cell death induced by the UA-NP and MV combination.

### 2.8. Synergistic Killing Effect of Oncolytic MV and UA-NP Combined Treatment on BT-474 and MDA-MB-468 Breast Cancer Cells

**Figure 8:**
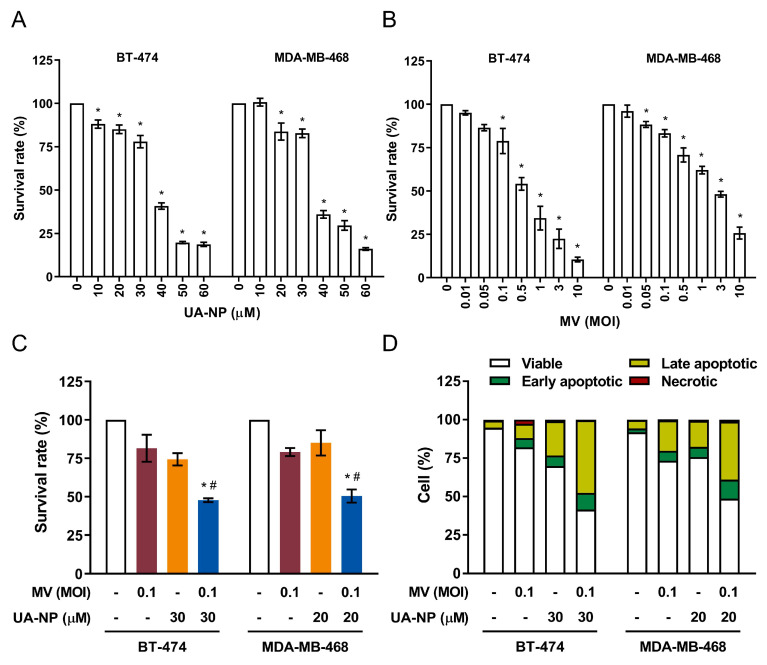
Combination treatment using UA-NP and oncolytic MV exerts enhanced anticancer effect against human breast cancer BT-474 and MDA-MB-468 cells. BT-474 and MDA-MB-468 cells were treated with (**A**) UA-NP (10–60 μM) or (**B**) MV (MOI 0.01–10) for 5 days before analysis of cell viability using MTT assay. DMSO = 0.1%. (**C**) BT-474 and MDA-MB-468 cells were co-treated MV (MOI 0.1) and/or UA-NP (30 μM for BT-474 and 20 μM for MDA-MB-468), following which cell viability was determined by MTT assay. (**D**) BT-474 and MDA-MB-468 cells were first concurrently treated with UA-NP (30 or 20 µM) and MV (MOI 0.1) for 5 days, and then analyzed by flow cytometry for apoptosis induction. Data shown are means ± SEM from three independent experiments; * *p* < 0.05 in (**A**,**B**); * *p* < 0.05 compared with MV MOI 0.01 or 0.1, ^#^ *p* < 0.05 compared with UA treatment only in (**C**).

Lastly, we examined whether the combined treatment using oncolytic MV and UA-NP could also exert enhanced killing effect on other breast cancer cell lines. To this end, we tested each agent, alone or their combination, in the human breast cancer BT-474 and MDA-MB-468 cells. While MCF-7 represents luminal type A breast cancer (ER+, PR+/−, HER2-), BT-474 represents luminal type B (ER+, PR+/−, HER2+) [33], and MDA-MB-468 is a triple-negative breast cancer (TNBC) cell line [34]. Both BT-474 and MDA-MB-468 are known to express Nectin-4 [17,24], which is used by MV to enter the host cell [24,35]. As shown in Figure 8, both UA-NP (Figure 8A) and oncolytic MV (Figure 8B) induced a dose-dependent cytotoxic effect on BT-474 and MDA-MB-468 cells. Using the UA-NP threshold concentration as mono-agent producing > 50% viable cells for BT-474 (30 µM) and MDA-MB-468 (20 µM), its combination with MOI 0.1 of oncolytic MV caused significant increased cell death in both cell lines compared to each agent alone (Figure 8C). Similar to MCF-7 cells, this enhanced killing effect in both cell lines appeared to be synergistic (CI = 0.9 for BT-474 and 0.6 for MDA-MB-468). Increased apoptosis was also observed in both cell lines when treated with the MV and UA-NP combination (Figure 8D). Cell viability analyses using LDH release also corroborated these results (Supplementary Figures S2 and S3).

Altogether, our results demonstrated that UA-NP and oncolytic MV could be used in combination to induce an enhanced cell death in the breast cancer cells.

## 3. Discussion

Treatment toxicities and limited efficacy against metastases in the current standard breast cancer therapies, particularly the chemotherapeutics, have raised longstanding concerns. Most first-line drugs for breast cancer are known to cause severe adverse effects, such as taxane-induced peripheral neuropathy, anthracyclines- or trastuzumab-induced cardiotoxicity, drug-induced alopecia, everolimus-induced mucositis, CDK4/6 inhibitor-induced neutropenia and diarrhea, PARP inhibitor-induced neutropenia and anemia, and receptor tyrosine kinase inhibitor-induced diarrhea [36]. Therefore, there is a critical need for developing new treatment modalities with reduced toxicity and improved efficacy, particularly against advanced and metastatic breast cancer. Oncolytic virotherapy is one such novel modality, and with most oncolytic viruses having limited clinical efficacy as single agents, combinatorial treatment with anticancer agents is thus frequently explored. Our discovery of the synergistic activity produced from the combination of oncolytic MV with the anticancer agent UA provides the alternate strategy of using a chemovirotherapeutic approach that offers enhancement of anticancer effect while reducing the agents’ dose, and hence would reduce toxicity to normal cells [23]. In addition, the use of oncolytic virotherapy offers additional advantages, given its high specificity against tumor cells due to viral tropism and thus low off-target toxicities, as well as the systemic effect of the oncolytic virus which facilitates detection and elimination of distant micro- and macrometastases [3]. Of course, effective in vivo doses of UA, UA-NP, and MV depend on multiple pharmacokinetic factors including the agent’s half-life, plasma area under the curve (AUC) value, toxicity, infusion route and infusion schedule, etc. and remain to be investigated further. Nonetheless, from the observation of synergized anticancer effect, we would expect lowered and thus more achievable doses of UA and MV in combination treatment as compared to their mono-agent therapies, which, in turn, would also likely reduce the toxicities associated with each agent. On the other hand, although not explored in the current study, resistance to UA has been reported in HT-29 colorectal cancer cells and DU145 prostate cancer cells [37], and resistance to oncolytic MV has also been observed in several cancer cell lines [38]. Whether the UA/UA-NP and MV combination could revert such resistance phenotype is worth examining due to the tumor heterogeneity in real life.

Individually, both oncolytic MV and UA have significant potential against breast cancer. The newly discovered oncolytic MV receptor and tumor marker Nectin-4 is selectively overexpressed and identified as an important therapeutic target for primary and metastatic triple-negative breast cancers as well as for lung, bladder, and pancreatic cancers [14,16,17,39,40,41], which makes Nectin-4-specific oncolytic MV a crucial novel tool in targeting Nectin-4-positive breast cancer and other adenocarcinomas. UA, on the other hand, is also a valuable and potent anticancer agent that exerts robust inhibitory activity against different breast cancer cell types in vitro and in vivo [10]. Both wild type MV infection and UA have been reported to induce G0/G1 cell cycle arrest and thus apoptotic cell death at the concentrations used in this study [42,43,44]. Our findings demonstrated the same pro-apoptotic effect of MV and UA on MCF-7 breast cancer cells, which was significantly enhanced through MV and UA co-treatment (Figure 3), leading us to speculate possible reinforcement of their individual anticancer effect when used in combination. Indeed, our findings reveal that oncolytic MV and UA combinatorial treatment exerts synergistic anti-breast-cancer efficacy (Figure 1), demonstrating that UA potentiates oncolytic MV-mediated killing of breast cancer cells. Despite past records of antiviral activity of UA against herpes simplex virus (HSV)-1 [45], human immunodeficiency virus (HIV) [46], coxsackievirus B1 (CVB1), and enterovirus 71 (EV71) [47], we did not observe any interference from UA on the oncolytic MV infection (Figure 2). It is possible that the antiviral activity from UA is specific to certain viruses. The absence of antagonism from UA on the oncolytic MV life cycle thus further supports their use in combined treatment. On the other hand, we confirmed that both UA-NP and MV independently induced autophagy in MCF-7 cells, and their combination further enhanced the autophagic flux (Figure 7), which is pro-viral for MV infection and its cytopathic effect [31]. These results could partially explain why the combination treatment enhanced cancer cell death more than either agent alone, although further in-depth investigation is required to fully illustrate the underlying mechanism(s).

Although UA has been considered as a potential anticancer drug, its solubility profile has severely limited its medicinal application. Specifically, crude UA’s solubility in water at 25 °C is about 2.2 × 10^−4^ µM [48], which is considerably below its anticancer CC_50_ concentration (16.67 ± 1.10 µM) against MCF-7 cells when completely dissolved in DMSO. In view of the caveats of poor water solubility and, hence, low bioavailability of UA, we have successfully generated nanoformulated UA (UA-NP) with improved drug dissolution profile. Biophysical examination by FESEM showed micrometer needle-shaped UA crystals that were rendered into the nanoscale UA particles (Figure 4A,B), an effect likely due to the impact of the emulsion-solvent diffusion with the water-soluble PVP that dispersed the UA crystals into an amorphous state, as evidenced by our XRD analysis (Figure 4C). Moreover, the amorphous UA in the UA-NP would be advantageous since lower energy is required in its dissolution process, as attested by the results obtained from the dissolution study (Figure 4D). These observations therefore support our formulation design and the hydrophilic PVP as beneficial strategies for improving UA’s dissolution profile. More importantly, the UA-NP retained their anticancer effect and could act synergistically with the oncolytic MV (Figure 5 and Figure 8), which was previously observed with non-formulated UA dissolved in DMSO. Given the known toxicity of DMSO solvent [49], the UA-NP therefore demonstrate an important advantage in future development of anticancer modalities involving UA.

## 4. Materials and Methods

### 4.1. Cell Culture, Virus and Reagents

Human breast adenocarcinoma cell lines MCF-7 (kindly provided by Dr. Ming-Sound Tsao; Ontario Cancer Institute, Toronto, ON, Canada), BT-474 (ATCC HTB-20; American Type Culture Collection, Rockville, MD, USA), and MDA-MB-468 (ATCC HTB-132; ATCC) were maintained in Dulbecco’s Modified Eagle medium (DMEM; GIBCO-Invitrogen; Carlsbad, CA, USA), supplemented with 10% fetal bovine serum (FBS; GIBCO-Invitrogen), 100 U/mL of penicillin G, 100 µg/mL of streptomycin, and 0.25 µg/mL of amphotericin B (GIBCO-Invitrogen) in a 5% CO_2_ humidified incubator at 37 °C. The recombinant Ichinose-B 323 wild type measles virus tagged with enhanced green fluorescent protein (MV.IC323-EGFP) was obtained from Dr. Roberto Cattaneo (Mayo Clinic, Rochester, MN, USA) and propagated in marmoset B lymphoblastoid cells (B95a) as previously described [24]. The 50% tissue culture infective dose (TCID_50_) assay was used to determine the viral titer, and virus concentrations were represented by MOI. Ursolic acid (UA) and polyvinylpyrrolidone (PVP) were purchased from Sigma-Aldrich Chemicals Co. (St. Louis, MO, USA). All other experimental reagents were of analytical grade.

### 4.2. Cell Viability Assay

The impact of treatment on breast cancer cell viability was assessed using the 3-(4,5-dimethylthiazol-2-yl)-2,5-diphenyltetrazolium bromide (MTT) cell viability assay kit (Merck-Millipore; Bedford, MA, USA). Briefly, cells seeded in 96-well plates (10^4^ cells per well) were treated with various concentrations of the test agent over a 5-day incubation at 37 °C. Cell viability was then determined according to the manufacturer’s protocol, and optical densities (OD) were recorded at 550 nm using an ELISA plate reader and calculated as follows: Cell viability (%) = (Absorbance _test compound_/Absorbance _control_) × 100%. The 50% cytotoxic concentration (CC_50_) value was then calculated using the GraphPad Prism 7 software (GraphPad Software; San Diego, CA, USA). Additional analysis using lactate dehydrogenase (LDH) cytotoxicity detection kit (Takara Bio; Kusatsu, Shiga, Japan) is described in the Supplementary Information.

### 4.3. Synergistic Effect of Drug-Virus Co-Treatment

To determine the impact of drug-virus co-treatment, cells seeded in 96-well plates (10^4^ cells per well) were infected with MV (MOI 0.01 or 0.1) and concurrently treated with drug. The MV infection was performed for 1.5 h at 37 °C, and cells were washed with phosphate buffered saline (PBS; HyClone, GE Healthcare, Chicago, IL, USA) before and after viral challenge. Subsequently, cells were incubated in fresh media containing the drug at 37 °C for 5 days. Cell viability was determined via the MTT or LDH assay as described above. The impact of drug-virus co-treatment on the cancer cell viability was determined by calculating the combination index (CI) value using the Chou–Talalay method with the equation CI = (D)_1_/(D_x_)_1_ + (D)_2_/(D_x_)_2_, where (D_x_)_1_, (D_x_)_2_ are the respective concentrations of drug 1 and drug 2 used in their single treatments that decrease the cell viability by x%, and (D)_1_, (D)_2_ are the respective concentrations of drug 1 in combination with drug 2 that together decreased the cell viability by x% [50]. Calculation of the CI value is easily done using the CompuSyn software developed by T. C. Chou and Nick Martin. The effect was determined as follows: additive (CI = 1), synergistic (CI < 1) or antagonistic (CI > 1) [50].

### 4.4. Influence of UA Treatment on Early Viral Entry Steps of MV Infection

The impact of UA treatment on the early MV viral entry steps, including (i) free virus particles, (ii) viral attachment, and (iii) viral penetration was examined as previously described [51] with some modifications. (i) Cell-free MV particles were first incubated with varying concentrations of UA (4, 5, 8 and 11 µM) for 3 h at 37 °C. The virus-drug mixture was then diluted 20-fold with DMEM containing 2% FBS to ineffective concentrations of UA and a final MV MOI of 0.1, before addition to MCF-7 seeded in 96-well plates (10^4^ cells per well) for 1.5 h. Cells were then washed with PBS and incubated in fresh 2% FBS DMEM for 3 days at 37 °C. (ii) The virus-drug inoculum containing MV (MOI 0.1) and varying concentrations of UA (2, 5 and 10 µM) were prepared and added to pre-chilled (at 4 °C) MCF-7 cells seeded in 96-well plates (10^4^ cells per well) for 1.5 h at 4 °C, before removing the virus-drug inoculum, washing with PBS, and further incubating for 3 days at 37 °C in fresh 2% FBS DMEM. (iii) MCF-7 cells seeded in 96-well plates (10^4^ cells per well) were first pre-bound with MV (MOI 0.1) at 4 °C for 1.5 h, which allows for virus binding but precludes internalization [52]. This was then followed by removal of viral inoculum and washing with PBS before shifting the temperature to 37 °C to facilitate viral penetration and treating the cells with varying concentrations of UA (2, 5 and 10 µM) for 1.5 h. The supernatant was subsequently removed and the wells were washed with PBS before incubation for 3 days at 37 °C in fresh 2% FBS DMEM. In all of the above, after the 3-day incubation, fluorescence signals from the reporter-tagged virus were scanned using the Typhoon 9410 variable mode imager (Amersham Biosciences; Baie d’Urfe, QC, Canada) and quantified using Image Quant TL software (Amersham Biosciences) to assess the viral infectivity.

### 4.5. Time-of-Drug-Addition Assays

Drug addition at different time-points (denoted as “pre-treatment”, “co-addition”, and “post-infection”) were performed as previously described [52] to investigate potential antiviral effect of UA on oncolytic MV infection of MCF-7 cells (10^4^ cells per well of 96-well plates). In pre-treatment analysis, cells were pre-treated with UA (2, 5, and 10 µM) for 24 h after which the supernatants were removed and cells were challenged with MV (MOI 0.1) for 1.5 h at 37 °C. Viral inoculum was later removed and replaced with fresh 2% FBS DMEM for 3 days’ incubation at 37 °C. In co-addition analysis, MV (MOI 0.1) and UA (2, 5, and 10 µM) were concurrently added to the cells for 1.5 h before the virus-drug inoculum was discarded and replaced with fresh 2% FBS DMEM for 3 days’ incubation at 37 °C. In post-infection analysis, cells were infected with MV (MOI 0.1) for 1.5 h, followed by removal of viral inoculum before incubating the cells with 2% FBS DMEM containing UA (2, 5 and 10 µM) for 3 days at 37 °C. For all assays, supernatants were collected at the end of the 3-day incubation for viral titration using TCID_50_.

### 4.6. Cell Cycle Analysis

Cells (3 × 10^5^ cells per well of 6-well plates) were treated with MV (MOI 0.01 or 0.1) and the drug, individually or concurrently, for 1.5 h at 37 °C. The supernatants were subsequently removed and the treated cells were refreshed with 2% FBS DMEM with or without the drug for 5 days’ incubation at 37 °C. The cells were then trypsinized, collected into 15 mL tubes, washed twice with ice-cold PBS by centrifugation, and finally fixed with 70% ethanol overnight at 4 °C. After fixation, the cells were washed twice with PBS by centrifugation prior to 30 min incubation in PBS solution containing 10 mg/mL ribonuclease A from bovine pancreas (RNase A; Sigma-Aldrich) at 37 °C. Propidium iodide (PI; 40 µg/mL; Sigma-Aldrich) was subsequently added to the cells for 15 min incubation in the dark at 37 °C before subjecting to flow cytometric cell cycle analysis using the Beckman Coulter FC500 flow cytometer (Beckman Coulter Inc.; Brea, CA, USA).

### 4.7. Apoptosis Analysis by Annexin V/Propidium Iodide Double Staining

Cells (3 x 10^5^ cells per well) seeded in 6-well plates were treated with MV (MOI 0.01 or 0.1) and the drug, individually or concurrently, for 1.5 h at 37 °C. The supernatants were then removed before refreshing the cells with 2% FBS DMEM with or without the drug for 5 days’ incubation at 37 °C. Subsequently, the cells were trypsinized for collection into 15 mL tubes, ice-cold PBS-washed twice by centrifugation, and finally resuspended in binding buffer containing 1 µl/mL PI and 1 µl/mL allophycocyanin (APC)-conjugated Annexin V (Enzo Life Sciences, Inc.; East Farmingdale, NY, USA). Apoptosis detection via flow cytometry was then performed using the Beckman Coulter FC500 apparatus (Beckman Coulter Inc.).

### 4.8. Western Blot Analysis

For PARP analysis, cells (3 × 10^5^ cells per well) seeded in 6-well plates were treated with MV (MOI 0.01 or 0.1) and the drug, individually or concurrently, for 1.5 h at 37 °C. The virus-drug inoculum was then discarded and the cells were refreshed with 2% FBS DMEM with or without the drug for a 5-day incubation at 37 °C. The cells were subsequently lysed with radioimmunoprecipitation assay (RIPA) buffer (Sigma-Aldrich) containing protease inhibitor (Roche Molecular Biochemicals; Indianapolis, IN, USA), and protein concentrations were measured by bicinchoninic acid (BCA) protein assay kit (Thermo Fisher Scientific Inc.; San Jose, CA, USA). Protein samples were then subjected to standard western blot analysis and proteins were probed using primary antibodies for PARP (1:1000; Cell Signaling Technology, Inc., Danvers, MA, USA) and β-actin (1:10,000; Cell Signaling Technology, Inc.) followed by anti-rabbit and anti-mouse horseradish peroxidase (HRP)-conjugated secondary antibodies (1:1000 and 1:10,000; Cell Signaling Technology, Inc.). For autophagy analysis, cells seeded in 12-well plates (2.5 × 10^5^ cells per well) were treated with UA-NP (30 μM), MV (MOI 0.1), or concurrently treated with both agents for 48 h before being harvested and analyzed for LC3 (1:1000; Thermo Fisher Scientific), p62 (1:1000; GeneTex, Irvine, CA, USA), and β-actin (1:10,000; Cell Signaling Technology) expression using western blotting. Bafilomycin A1 (BAF, 100 nM; Sigma-Aldrich) was added to the indicated groups 4 h before harvesting the cells. MV H protein was probed to indicate MV infection using a rabbit anti-MV H serum H606 [53] (1:1000; kindly provided by Dr. Christian Buchholz; Paul-Ehrlich-Institut, Langen, Germany). Detection was performed using the Immobilon™ Western Chemiluminescent HRP substrate (Merck Millipore; Burlington, MA, USA), followed by chemiluminescence imaging using the UVP BioSpectrum 500 imaging system (UVP; Upland, CA, USA). Protein band intensities were quantitatively evaluated and compared against the β-actin loading control via densitometry analysis.

### 4.9. Preparation of UA Nanoparticles (UA-NP)

The UA loaded nanoparticles (UA-NP) were prepared using the emulsion-solvent diffusion technique as previously described [54]. UA was first dissolved in ethanol to obtain an organic phase (UA concentration = 3 mg/mL), which was then mixed with the aqueous PVP (prepared in water) at 1:1, 1:3 or 1:6 UA to PVP weight ratios. Subsequently, the solutions were homogenized by sonication at 20 kHz for 10 min in a cold-water bath. Next, the organic ethanol solvent was removed using a rotary vacuum evaporator with water bath at 40 °C. The residue was passed through qualitative filter paper (Advantec^®^ No. 1, Toyo Roshi Kaisha, Ltd., Tokyo, Japan) to remove aggregates. The filtrate containing UA-NP was collected and stored at 4 °C for immediate use, or lyophilized and then stored at −20 °C in a moisture-proof container for longer period storage.

### 4.10. UA-NP Particle Size Analysis by Photon Correlation Spectroscopy (PCS)

Mean size of UA-NP was measured by the Malvern Zetasizer (Malvern Instruments Ltd., Worcestershire, United Kingdom). The temperature was maintained at 25 °C and the test sample was diluted 50-fold using deionized water before measurement. Each determination was performed in triplicate.

### 4.11. High-Performance Liquid Chromatography (HPLC) Analysis of UA and UA-NP

HPLC was performed using the Hitachi D-7000 HPLC system (Hitachi, Ltd.; Tokyo, Japan) with a reverse-phase C18 column (LichroCART^®^ Purospher^®^ STAR; Merck KGaA; Darmstadt, Germany). The mobile phase consisted of acetonitrile and 0.1% phosphoric acid (85:15, *v/v*). The analytical process was carried out under a flow rate of 1 mL/min for 15 min with UV detection at 210 nm. The calibration curve was linear over the concentration range of 0.45–90 μg/mL with a coefficient estimate of 0.999.

### 4.12. UA-NP Yield Quantification

The yield of UA nanoformulation was determined by HPLC analysis using a previously described method [54]:

Yield (%) = (C_UA_ (μg/mL) × V_UA-NP_ (ml)/W_UA_ (μg)) × 100

C_UA_ (μg/mL): concentration of UA detected in UA-NP

V_UA-NP_ (ml): final volume of UA-NP

W_UA_ (μg): quantity of UA used in the preparation for nanoformulation

### 4.13. Field Emission Scanning Electron Microscopy (FESEM)

The morphological characteristics of the UA-NP were imaged using FESEM as previously reported [54]. UA-NP samples were sputter-coated with gold in low energy input (E-1045 ion sputter; Hitachi, Ltd.; Tokyo, Japan) and then viewed under the Hitachi SU8010 SEM with an accelerating voltage of 5 kV.

### 4.14. X-ray Diffraction (XRD)

An X-ray diffractometer with Cu-Kα radiation and Ni filter (Siemens D5000; Siemens AG; Munich, Germany) was used to examine the crystalline properties of UA, PVP, UA-PVP physical mixture (UA-PM), and lyophilized UA-NP powder. UA-PM was prepared by thoroughly mixing UA and PVP with a mortar in the same composition ratio as for UA-NP, which was 1:1 in weight. Prior to XRD analysis, all samples were dried overnight to remove moisture. XRD patterns were obtained at 40 kV and 25 mA, with a scanning rate of 4°/min, over the diffraction angle (2θ) range of 2° to 50°.

### 4.15. Dissolution Test

The dissolution test was performed based on the United States Pharmacopeia (USP) apparatus II (paddle) method [54] in accordance to USP XXIX [55]. To achieve better discriminating dissolution profiles for the poorly soluble crude drug and its nanoformulation, non-sink condition was applied for the analysis [56,57]. Briefly, UA or UA-NP sampling powders were prepared by placing 4.68 mg equivalent of UA in 100 mL of pH 7.4 phosphate buffer with continuous stirring by paddle at 100 rpm and temperature maintained at 37 ± 0.5 °C (*n* = 6). During the experiment, 1 mL of sample was withdrawn at successive time intervals (0, 1, 10, 20, 30, 60, 90 and 120 min). The concentration of UA was analyzed by HPLC and the data acquired were calculated and converted into “percent of dissolved amount” to represent the drug dissolution property.

### 4.16. Statistical Analysis

All data are expressed as means ± the standard error of means (SEM) unless otherwise indicated. Statistical significance was evaluated using GraphPad Prism (GraphPad Software), by one-way analysis of variance (ANOVA) followed by Dunnett’s multiple comparison, and a *p* < 0.05 was considered statistically significant.

## 5. Conclusions

In conclusion, we demonstrated for the first time that UA and nanoformulated UA-NP can work synergistically with oncolytic MV in combinatorial treatments against breast cancer cells, by effectively enhancing induction of apoptotic cell death, thus facilitating improved anticancer efficacy as compared to mono-treatment with either agent alone. Our results also showed that combination of UA-NP with MV exerts synergistic killing effect against various breast cancer cell types, including luminal type A, luminal type B, and TNBC. This observation indicates that oncolytic MV chemovirotherapy using UA-based combinations merit further investigation and development as a treatment strategy for the management of breast cancers. Future studies should also explore whether such combinations would also be effective against other Nectin-4-positive adenocarcinomas.

## Data Availability

Data is contained within the article or supplementary material.

## Supplementary Materials

The following are available online at https://www.mdpi.com/2072-6694/13/1/136/s1, Figure S1: LDH release assay to assess cell viability of UA-NP and oncolytic MV co-treatment in MCF-7 human breast cancer cells, Figure S2: LDH release assay to assess cell viability of UA-NP and oncolytic MV co-treatment in BT-474 human breast cancer cells, Figure S3: LDH release assay to assess cell viability of UA-NP and oncolytic MV co-treatment in MDA-MB-468 human breast cancer cells, Figure S4: Full-length blots.
