# Supplementary material for: Ursolic Acid and Its Nanoparticles Are Potentiators of Oncolytic Measles Virotherapy against Breast Cancer Cells
Source: Cancers (Basel). 2021 Jan 4;13(1):136. doi: 10.3390/cancers13010136 (PMC7795983; doi:10.3390/cancers13010136)
Supplement: Supplementary file 1 [file cancers-13-00136-s001.pdf]

Supplementary Figure S1. LDH release assay to assess cell viability of UA-NP and oncolytic MV co-treatment in MCF-7 human breast cancer cells.

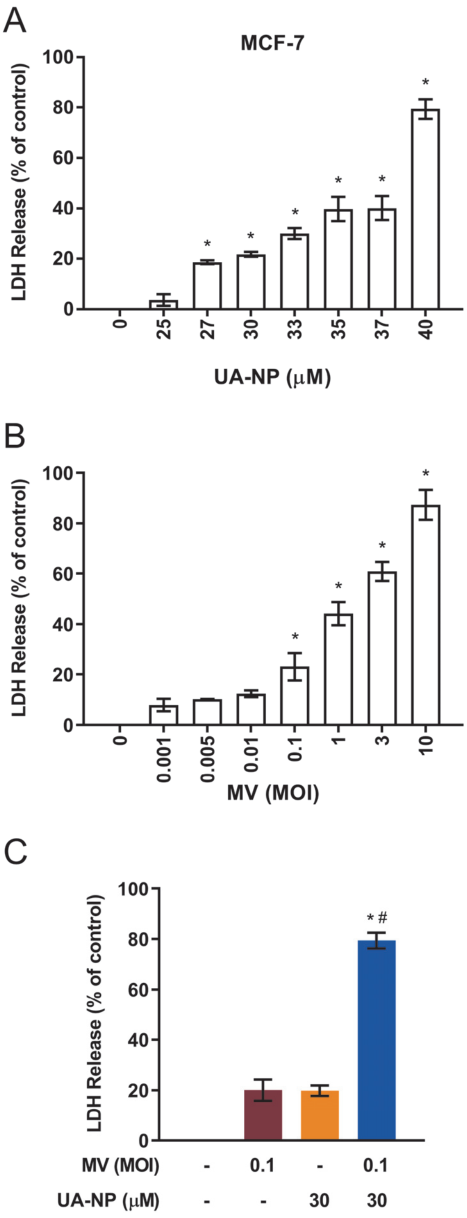

MCF-7 cells seeded in 96-well plates (10^4 cells per well) were treated with (**A**) UA-NP (25–40 μM), (**B**) MV (MOI 0.001–10), or (**C**) concurrently treated with MV (MOI 0.1) and/or UA-NP (30 μM) for 5 days before analyzing cell viability using lactate dehydrogenase (LDH) cytotoxicity detection kit (Takara Bio; Kusatsu, Japan) following the manufacturer's instructions. Briefly, supernatant was transferred to new 96-well plates, mixed with reaction substrates, and incubated at 37 °C for 30 min before the absorbance was measured at 490 nm using an ELISA plate reader. All data shown are means ± SEM (* $p < 0.05$ in panels **A** and **B**; * $p < 0.05$ compared with MV, $^{\#}$ $p < 0.05$ compared with UA treatment in panel **C**) from three independent experiments.

Supplementary Figure S2. LDH release assay to assess cell viability of UA-NP and oncolytic MV co-treatment in BT-474 human breast cancer cells.

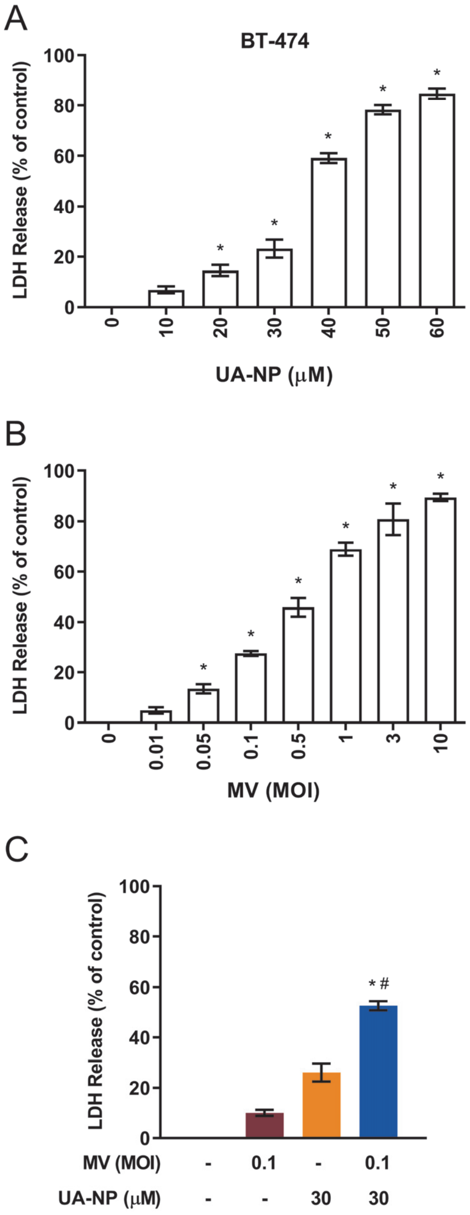

BT-474 cells seeded in 96-well plates (10^4 cells per well) were treated with (**A**) UA-NP (10–60 μM), (**B**) MV (MOI 0.01–10), or (**C**) concurrently treated with MV (MOI 0.1) and/or UA-NP (30 μM) for 5 days before analyzing cell viability using LDH cytotoxicity detection kit (Takara Bio) as described in Supplementary Figure S1. All data shown are means ± SEM (* $p < 0.05$ in panels **A** and **B**; * $p < 0.05$ compared with MV, $^{\#}$ $p < 0.05$ compared with UA treatment in panel **C**) from three independent experiments.

Supplementary Figure S3. LDH release assay to assess cell viability of UA-NP and oncolytic MV co-treatment in MDA-MB-468 human breast cancer cells.

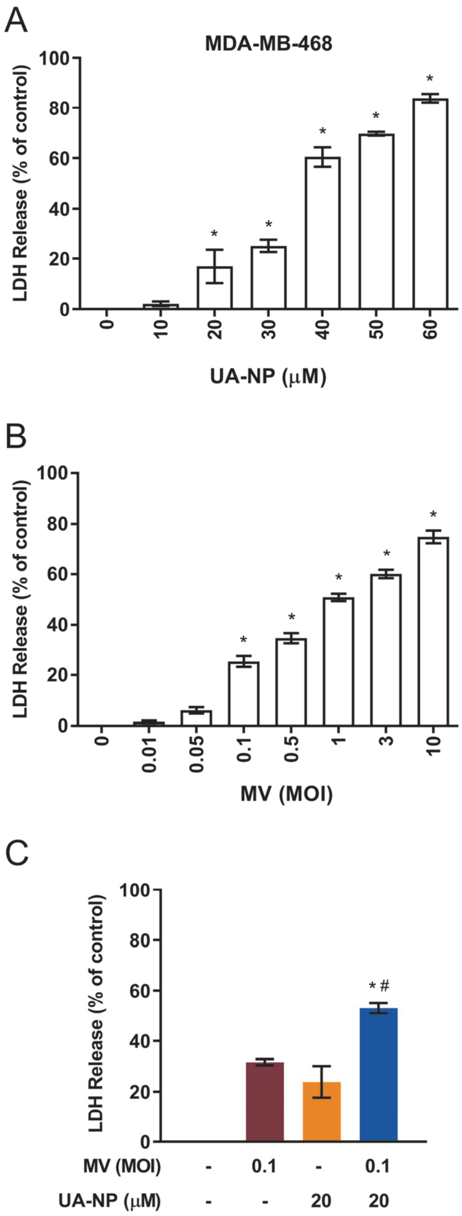

MDA-MB-468 cells seeded in 96-well plates (10^4 cells per well) were treated with (**A**) UA-NP (10–60 μM), (**B**) MV (MOI 0.01–10), or (**C**) concurrently treated with MV (MOI 0.1) and/or UA-NP (20 μM) for 5 days before analyzing cell viability using LDH cytotoxicity detection kit (Takara Bio) as described in Supplementary Figure S1. All data shown are means ± SEM (* *$p < 0.05$* in panels **A** and **B**; * *$p < 0.05$* compared with MV, $^{\#}$ *$p < 0.05$* compared with UA treatment in panel **C**) from three independent experiments.

Supplementary Figure S4. Full-length blots.

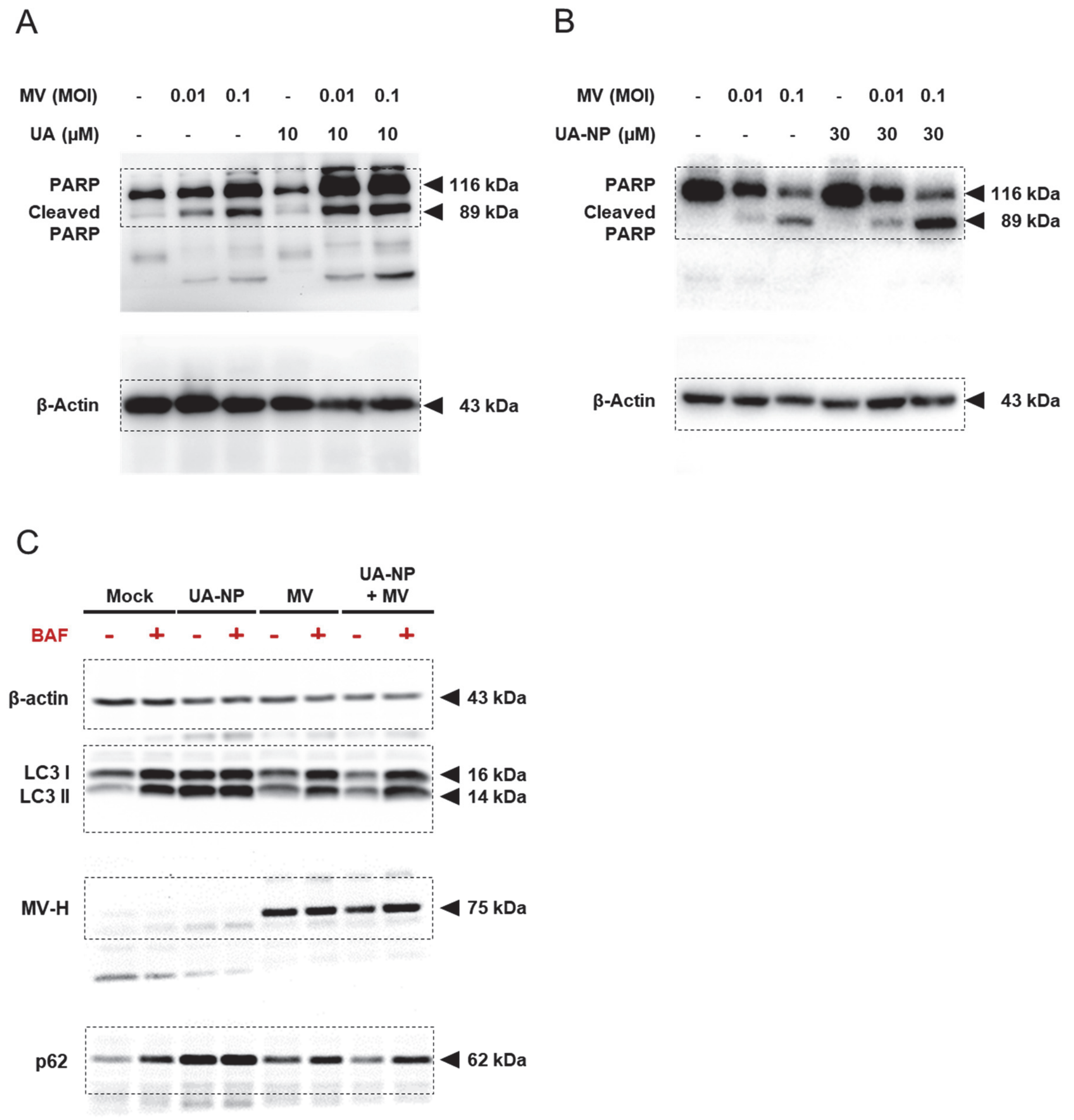

Full-length blots: (**A**) Figure 3C, (**B**) Figure 6C, and (**C**) Figure 7.
